# Molecular Recognition Insights of Sialic Acid Glycans by Distinct Receptors Unveiled by NMR and Molecular Modeling

**DOI:** 10.3389/fmolb.2021.727847

**Published:** 2021-11-15

**Authors:** Cátia Oliveira Soares, Ana Sofia Grosso, June Ereño-Orbea, Helena Coelho, Filipa Marcelo

**Affiliations:** ^1^ Associate Laboratory i4HB–Institute for Health and Bioeconomy, NOVA School of Science and Technology, NOVA University Lisbon, Caparica, Portugal; ^2^ Department of Chemistry, UCIBIO–Applied Molecular Biosciences Unit, NOVA School of Science and Technology, NOVA University Lisbon, Caparica, Portugal; ^3^ CIC bioGUNE, Basque Research and Technology Alliance, Bizkaia Technology Park, Bilbao, Spain; ^4^ Ikerbasque, Basque Foundation for Science, Bilbao, Spain

**Keywords:** sialic-acid, siglecs, virus, bacteria, Nuclear Magnetic Resonance, molecular recognition

## Abstract

All cells are decorated with a highly dense and complex structure of glycan chains, which are mostly attached to proteins and lipids. In this context, sialic acids are a family of nine-carbon acidic monosaccharides typically found at the terminal position of glycan chains, modulating several physiological and pathological processes. Sialic acids have many structural and modulatory roles due to their negative charge and hydrophilicity. In addition, the recognition of sialic acid glycans by mammalian cell lectins, such as siglecs, has been described as an important immunological checkpoint. Furthermore, sialic acid glycans also play a pivotal role in host–pathogen interactions. Various pathogen receptors exposed on the surface of viruses and bacteria are responsible for the binding to sialic acid sugars located on the surface of host cells, becoming a critical point of contact in the infection process. Understanding the molecular mechanism of sialic acid glycans recognition by sialic acid-binding proteins, present on the surface of pathogens or human cells, is essential to realize the biological mechanism of these events and paves the way for the rational development of strategies to modulate sialic acid-protein interactions in diseases. In this perspective, nuclear magnetic resonance (NMR) spectroscopy, assisted with molecular modeling protocols, is a versatile and powerful technique to investigate the structural and dynamic aspects of glycoconjugates and their interactions in solution at the atomic level. NMR provides the corresponding ligand and protein epitopes, essential for designing and developing potential glycan-based therapies. In this review, we critically discuss the current state of knowledge about the structural features behind the molecular recognition of sialic acid glycans by different receptors, naturally present on human cells or pathogens, disclosed by NMR spectroscopy and molecular modeling protocols.

## Introduction

**FIGURE 1:**
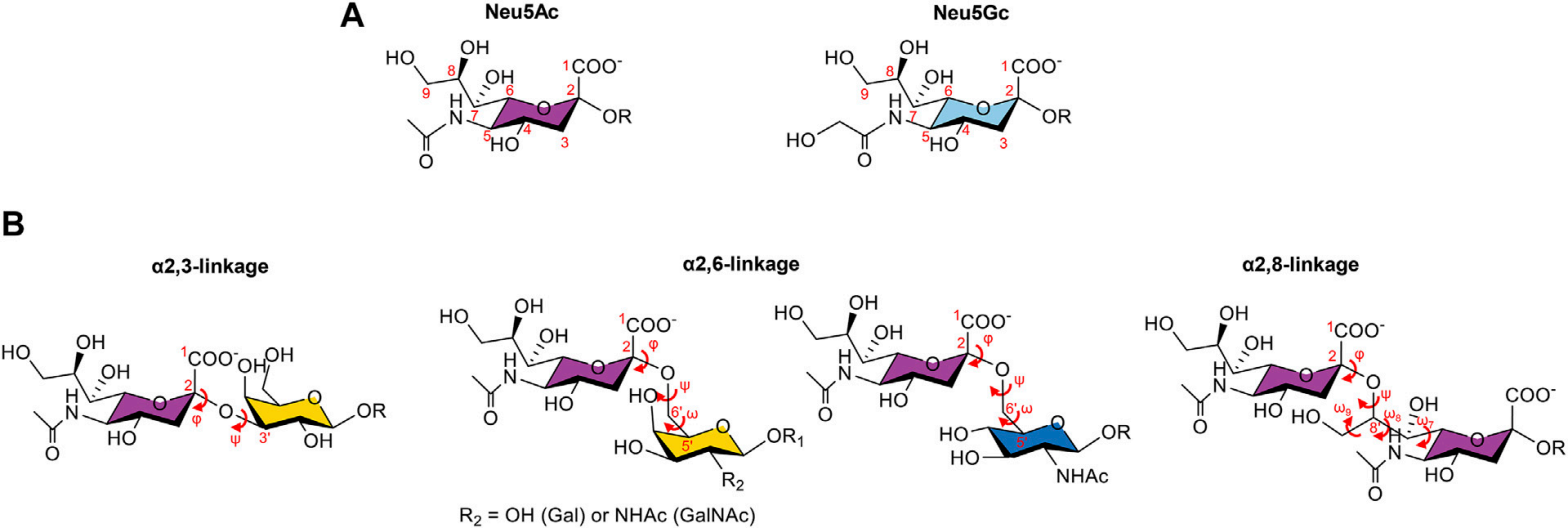
Two most common forms of sialic acids and the three types of glycosidic linkages observed in sialoglycans. **(A)**. Chemical structures of the *N*-acetylneuraminic acid (Neu5Ac) and the *N*-glycolylneuraminic acid (Neu5Gc), with the numbering of the carbon atoms in red. The difference lies in the group attached to C5: a *N*-acetyl group for Neu5Ac, which can be hydroxylated to form the *N*-glycolyl group of Neu5Gc. **(B)**. Representation of the α2,3-, α2,6-, and α2,8- linkages and the corresponding dihedral angles in red. Neu5Ac can be attached to a galactose (Gal) residue through the α2,3-linkage, described by the φ (C1-C2-O-C3′) and ψ (C2-O-C3′-H3′) torsion angles; to a Gal, a *N*-acetylgalactosamine (GalNAc), or a *N*-acetylglucosamine (GlcNAc) residue by an α2,6-linkage, characterized by φ (C1-C2-O-C6′), ψ (C2-O-C6′-C5′), and ω (O-C6′-C5′-O5′) torsion angles; or to another sialic acid, through an α2,8-linkage defined by the torsion angles φ (C1-C2-O-C8′), ψ (C2-O-C8′-C7′), ω9 (O9′-C9′-C8′-O), ω8 (O8′-C8′-C7′-O7′), and ω7 (O7′-C7′-C6′-O6′).

Sialic acids are nine-carbon monosaccharides constituted by a carboxylate group (C1) attached to a quaternary anomeric carbon (C2), a deoxygenated C3, an exocyclic 3-carbon glycerol side chain at C6, and different substituents at C5 (Figure 1A) (Schnaar et al., 2014; Varki et al., 2015). The most common forms of sialic acids in nature are the *N*-acetylneuraminic acid (Neu5Ac), which is the most abundant form in humans, and the *N*-glycolylneuraminic acid (Neu5Gc) (Traving and Schauer, 1998). This last form can also be found in human cells; however, its source is exogenous since humans cannot biosynthesize it due to an inactivating deletion in the CMAH gene encoding the CMP-Neu5Ac hydroxylase enzyme, and therefore, its presence is often associated with pathological processes (Chou et al., 1998; Traving and Schauer, 1998; Schauer and Kamerling, 2018).

The family of sialic acid glycans (sialoglycans) is extremely diverse and dynamic (Schauer and Kamerling, 2018). Their diversity is associated with the type of linkages (the α2,3-, α2,6-, and α2,8- linkages) in which the sialic acids can participate with the underlying glycan to which they can be attached and also with different modifications that can occur on the hydroxyl groups at positions 4, 7, 8, and 9 (O-acetylation, O-methylation, O-lactylation, O-sulfanation, and O-phosphorylation), enabling the existence of more than 80 derivatives of sialic acids (Cohen and Varki, 2010; Schauer and Kamerling, 2018) (Figure 1B).

The physical properties of sialic acids (negatively charged and hydrophilic), as well as their location, which commonly appear as terminal sugar moieties of many glycoconjugates (glycolipids and glycoproteins), make them key recognition sites not only for several human physiological receptors (such as selectins and siglecs) but also for toxins and receptors present in pathogens (such as viruses and bacteria) (Varki et al., 2015). The specificity of these molecular recognition events is modulated by the conformation of the sialoglycan, which strongly influences the presentation of the Neu5Ac residue to the receptor, and by additional interactions established by other sugar residues and functional groups present in the sialoglycan and in the receptor (Veluraja et al., 2010). Therefore, to fully understand these processes, it is necessary to decipher the conformations and dynamics of these oligosaccharides in the free and receptor-bound states. Sialoglycans are characterized to be highly dynamic and flexible in solution, commonly originating multiple conformations in equilibrium (Veluraja et al., 2010). Hence, NMR spectroscopy assisted with computational methods (such as molecular mechanics, molecular dynamics, and Monte Carlo simulation) has proved to be a powerful and robust methodology to disentangle the conformations of sialic acid oligosaccharides in solution, as well as to unveil the molecular determinants that govern the interactions between sialic acids and receptors.

In this context, the present review is focused on the current knowledge of Neu5Ac-based sialoglycan’s conformation (*Conformation of Neu5Ac Sialoglycans in Solution*), together with their binding mechanisms to different receptors, naturally present in human cells (siglecs—*Sialic Acid–Siglec Interactions*) and on pathogens, namely, viruses (*Sialic Acid–Virus Interactions*) and bacteria (*Sialic Acid–Bacteria Interactions*), mainly disclosed by NMR spectroscopy and molecular modeling protocols.

## Conformation of Neu5Ac Sialoglycans in Solution

The diversity of the linkages through which Neu5Ac can be attached to a glycan chain dictates the conformation of the sialoglycan in solution and, consequently, influences its molecular interactions and affinities to different receptors. The most common glycosidic bonds involving Neu5Ac are the α2-3-linkage to a galactose (Gal) residue; the α2-6-linkage to a Gal, a *N*-acetylgalactosamine (GalNAc), or a *N*-acetylglucosamine (GlcNAc) residue; and the α2-8-linkage to another Neu5Ac residue (Figure 1B) (Cohen and Varki, 2010). The conformational analysis around the glycosidic linkages is discussed below in a more detailed manner. Nevertheless, it is important to notice that, apart from the flexibility around the glycosidic linkage, most of the studies show that the conformation of the glycerol side chain of the α2-Neu5Ac structure remains unchanged. The glycerol side chain is rigid, adopting an extended conformation in solution with dihedral angles H6-C6-C7-H7 and H7-C7-C8-H8 around −60° (-g conformer) and 180° (t conformer), respectively (Acquotti et al., 1991; Brocca et al., 2000). This conformation seems to be stabilized by a first H-bond between the OH8 and the carboxylic group, and a possible second H-bond between the OH7 and carbonyl group of the *N*-acetyl group. These H-bonds are experimentally supported by very small ^3^J_6,7_ (∼1 Hz) and fairly large ^3^J_7,8_ (∼8 Hz) vicinal coupling constants (Poppe et al., 1997) and by the Nuclear Overhauser Enhancement Spectroscopy (NOESY) analysis with the existence of a strong OH8-H6 NOE contact (Acquotti et al., 1994).

### Conformations Adopted by α2-3 Sialoglycans

A sialic acid forming an α2-3 linkage with a Gal residue is typically found in glycoproteins and glycolipids (Varki et al., 2015). Most conformational studies that have been done with α2-3 sialoglycans use the oligosaccharide sequences of gangliosides, sialylated Lewis antigens, and terminal glycan fragments of *O-* and *N-*glycoproteins (Poppe et al., 1994, Poppe et al., 1997; Brocca et al., 2000; Forgione et al., 2020a, Forgione et al., 2020b). Despite the variety of structures, the two glycosidic torsion angles around the Neu5Acα2-3Gal bond, φ (C1-C2-O-C3′) and ψ (C2-O-C3′-H3′), exhibit a similar behavior in most of the sialoglycans with this type of linkage. Normally, in solution, the φ value alternates among −60° (-g conformer), 180° (t conformer), and 60° (g conformer), while the ψ value remains stable. The population of each conformer in solution can be confirmed by the existence of the NOE cross peaks in the NOESY spectrum that are representative of each conformer. Specifically, the NOE H3_ax_ Neu5Ac–H3 Gal is compatible with the t conformer, the NOE H8 Neu5Ac–H3 Gal is consistent with the -g conformer, and the NOEs H3_eq_ Neu5Ac–H3 Gal and H3_eq_/H3_ax_ Neu5Ac–H4 Gal are the characteristics of the g conformer (Table 1) (Forgione et al., 2020a; Forgione et al., 2020b). All these conformers adopt an extended conformation, forming a cone-like topology (Xu et al., 2009; Forgione et al., 2020a).

**TABLE 1:** Possible conformers of an α2,3-sialoglycan in solution, represented by 3′SLN (Neu5Acα2-3Galβ1-4GlcNAc). These three conformers differ in the φ dihedral angle (C1-C2-O-C3′), which can adopt values of 180°, -60°, and 60° (t, -g, and g conformers, respectively), whereas the ψ dihedral angle (C2-O-C3′-H3′) remains stable at around −11°. The t and -g conformers are the most populated in solution. The information about each conformer and the corresponding NMR evidence proving their existence was retrieved from Forgione et al. (2020a) and Forgione et al. (2020b). The representative structures were generated using the carbohydrate builder tool from GLYCAM-web (Woods, 2005), and the images were created using PyMOL 2.4.1 (Schrödinger, 2010).

Φ(C1-C2-O-C3′)	180° (t conformer)	−60° (-g conformer)	60° (g conformer)
NMR Evidence	H3_ax_Neu5Ac-H3 Gal (Strong NOE)	H8 Neu5Ac-H3 Gal (Medium NOE)	H3_eq_ Neu5Ac-H3 Gal H3_eq_/H3_ax_ Neu5Ac-H4 Gal (Strong NOEs)
Representative structure	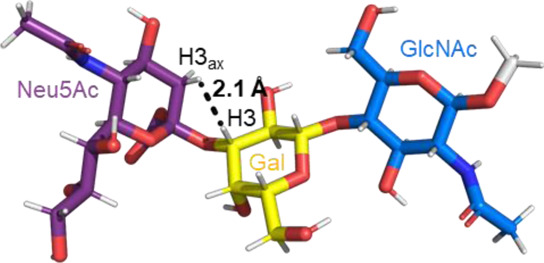	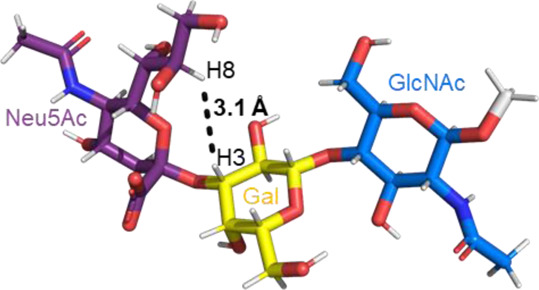	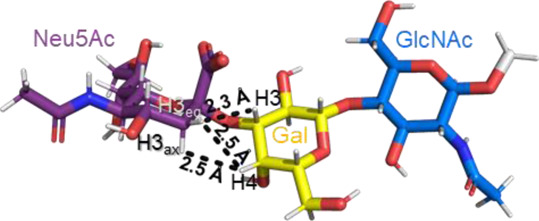

Remarkably, the major difference observed in the gangliosides (GM3/GM4 *vs* GM1/GM2) is related to whether the central Gal moiety is or is not substituted in OH4 with a GalNAc residue (Brocca et al., 2000). In general, the nonbranched gangliosides (GM3 and GM4) show higher flexibility in the Neu5Acα2-3Gal linkage than those 3,4 disubstituted at the central Gal residue, assuming two major conformers (t and -g) around the φ torsion angle (φ = 180° and -60°) in solution (Brocca et al., 2000). This same degree of freedom is also found in other α2-3 sialoglycans, such as in 3’sialyllactosamine (Neu5Acα2-3Galβ1-4GlcNAc, 3′SLN) and sialyl Lewis X (Neu5Acα2-3Galβ1-4(Fucα1-3)GlcNAc, sLe^x^) related structures (Brisson et al., 1997; Brocca et al., 2000). In particular, recent studies confirmed that the φ torsion angle of Neu5Acα2-3Gal linkage in the 3′SLN mainly explores values around −60° (-g conformer) and 180° (t conformer), while ψ remains stable around −11°C (Forgione et al., 2020a).

The branched gangliosides (GM1 and GM2), which encode a trisaccharide core constituted by GalNAcβ1-4(Neu5Acα2-3)Gal, also have two major populations of conformers. However, the conformation with the torsion angles φ and ψ assuming values of −160° and −25° (t conformer), respectively, is energetically favorable (80–90% of the total population) than that with φ/ψ values of −70°/-6° (-g conformer, 20–10% of the total population) (Brocca et al., 2000). This conformational preference favoring the t conformer seems to be strongly associated with the 3,4-disubstitution of the central Gal residue (Bernardi et al., 2002).

Interestingly, gangliosides with more than one sialic acid unit in their structure, such as GD1a and GT1b, have a major conformation for the internal trisaccharide core similar to that observed in the case of GM1/GM2 counterparts and multiple conformations for the external sialic linkage (Poppe et al., 1994; Venkateshwari and Veluraja, 2012). For GD1a, the external α2-3 linkage encodes both conformers with φ/ψ adopting 180°/0° and −60°/0°, supported by the co-existence of NOE contacts Neu5Ac H3_ax_–Gal H3 (φ = 180°) and Neu5Ac OH8/H8–Gal H3 (φ = −60°), while for the internal sialic linkage, a single conformer around the α2-3 linkage is deduced through the detection of the strong NOE contact Neu5Ac H3_ax_–Gal H3 along with the additional Neu5Ac OH8–Gal H4 NOE contact, therefore suggesting a φ/ψ around 180°/0° (Poppe et al., 1994). In the case of GT1b, the internal sialic linkage has two conformers in a 15:85 ratio with φ/ψ adopting −160°/30° and −70°/0°, respectively, while the external sialic linkage has three possible conformers populated at 9% (−150°/−50°), 65% (−90°/−60°), and 22% (−70°/−10°) (Venkateshwari and Veluraja, 2012).

Finally, modifications in the C9 and *N*-acetylation of the Neu5Ac only cause local modifications and do not induce changes in the torsion angles of the α2,3-glycosidic bond or any difference in the secondary structure (Li et al., 2020).

### Conformations Adopted by α2-6 Sialoglycans

The sialoglycans with an α2-6-linked Neu5Ac to a Gal, a GalNAc, or GlcNAc residue are less common than the α2-3-linked counterparts. Nevertheless, in cancer cells, some of these types of linkages become more prominent and can be found as terminal motifs of *N*-/*O*-glycans, as well as in glycolipids (Tanaka et al., 2010; Varki et al., 2015). In contrast to the α2-3 sialoglycans, the α2-6 ones do not suffer further modifications.

The Neu5Acα2-6-Gal linkage strongly influences the 3-D structure of the glycan in which it is present. Its conformation is modulated not only by the two torsional angles, φ (C1-C2-O-C6′) and ψ (C2-O-C6′-C5′) present in the glycosidic linkage, but also by the torsion angle ω (O-C6′-C5′-O5′) around the C5′-C6′ bond of Gal/GalNAc/GlcNAc, which offers additional flexibility to the linkage between the sialic acid and the Gal/GalNAc/GlcNAc unit, and the remaining backbone (Figure 1B). The conformational studies of the α2,6-sialyllactosamine structure (Neu5Acα2-6Galβ1-4GlcNAc, 6′SLN) revealed that the φ angle typically explores values around −60° (-g conformer) and 180° (t conformer) in solution, while the ψ angle remains constant around 180° (Di Carluccio et al., 2020; Forgione et al., 2020a) (Table 2). Nevertheless, the existence of a NOE contact between the H5 of Neu5Ac and the NHAc of GlcNAc indicates that the conformer holding φ around −60° is the major conformer in solution (Sassaki et al., 2013; Forgione et al., 2020a; Di Carluccio et al., 2020).

**TABLE 2:** Possible conformers of an α2,6-sialoglycan in solution, represented by 6′SLN (Neu5Acα2-6Galβ1-4GlcNAc). The φ dihedral angle (C1-C2-O-C6′) can adopt values of 180° and −60° (t and -g conformers, respectively), whereas the ω torsion angle (O-C6′-C5′-O5’) can explore three values, 180°, 60°, and −60° (tg, gt, and gg conformers, respectively). The ψ dihedral angle (C2-O-C3′-H3′) remains stable at around 180°. The information about each conformer and the corresponding NMR evidence proving their existence was retrieved from Forgione et al. (2020a) and Di Carluccio et al. (2020). The representative structures were generated using the Carbohydrate Builder tool from GLYCAM-web (Woods, 2005), and the images were created using PyMOL 2.4.1 (Schrödinger).

Φ(C1-C2-O-C6′)	180° (t conformer)	-60° (-g conformer)	
NMR evidence	H3_ax_Neu5Ac-H6 R/S Gal (Strong/Medium NOE)	H5 Neu5Ac-CH_3_ GlcNAC (Weak NOE)	
Representative structure	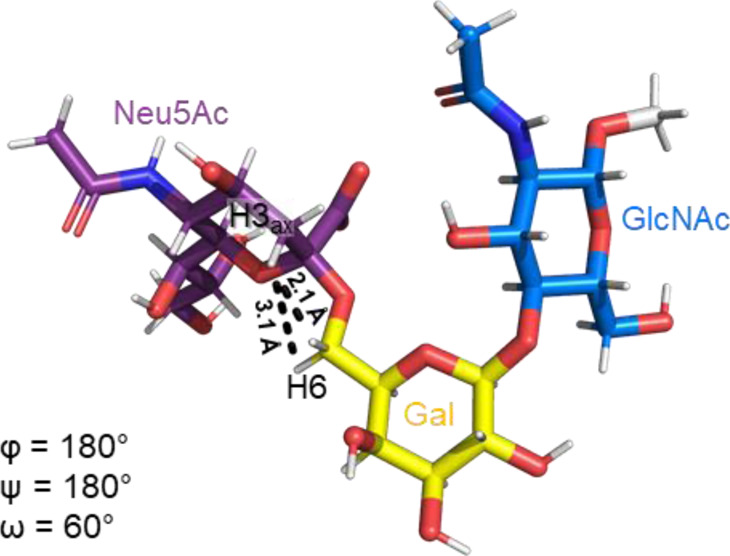	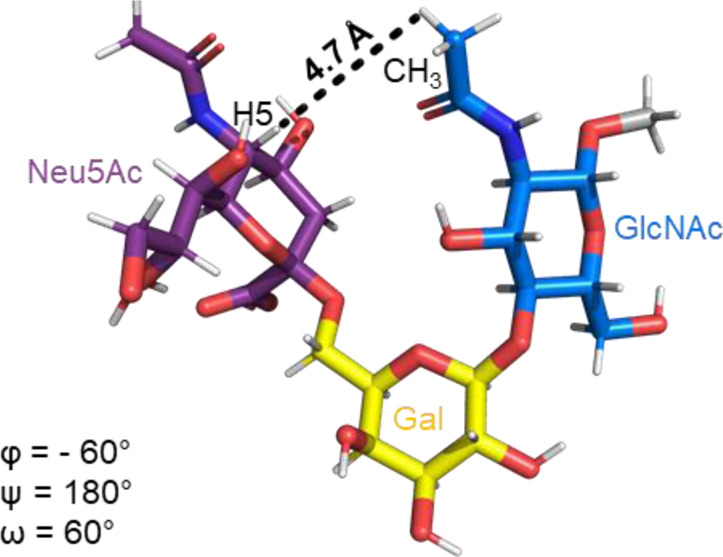	
ω(O-C6′-C5′-O5′)	180° (tg conformer)	60° (gt conformer)	−60° (gg conformer)
NMR evidence	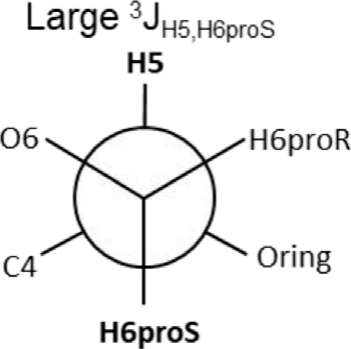	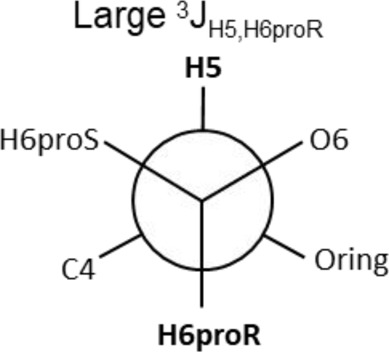	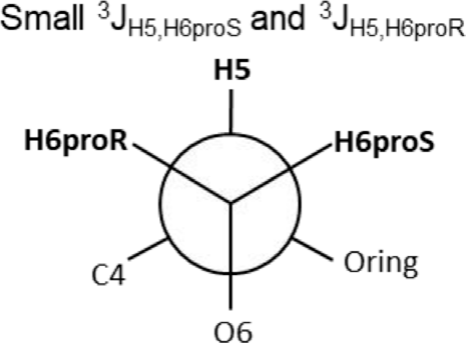

Additionally, the value of the ω dihedral angle that can adopt −60°/180°/60° corresponding to gg/tg/gt rotamers, respectively, has shown values around 60° (gt conformer) (Di Carluccio et al., 2019) (Table 2). In summary, for 6′SLN, the gt conformer (φ/ψ/ω of −60°/180°/60°) seems to be the most populated in solution and seems to be stabilized by intra-ligand van der Waals interactions and through H-bonds between Neu5Ac and GlcNAc. This conformation leads the 6′SLN to adopt an umbrella-like topology (bent conformation), defined by the angle between the carbon C2 of Neu5Ac and C1 atoms of the Gal and GlcNAc residues, which generally presents a value <110° (Table 2) (Sassaki et al., 2013; Forgione et al., 2020a; Di Carluccio et al., 2020). Interestingly, for the Neu5Gc derived trisaccharide, the absence of NOEs between the H6proR of Gal and the H3_ax_/H3_eq_ protons of Neu5Gc together with the existence of the NOE between the methyl group of NHAc of GlcNAc and H5 of Neu5Gc suggests that only the bent conformation (φ/ψ/ω of −60°/180°/60°) is present in solution (Di Carluccio et al., 2020).

The α2-6-linked Neu5Ac to a GalNAc is found in the STn antigen (Neu5Acα2-6GalNAcα-Thr/Ser), which is widely overexpressed in cancer cells as a common motif of aberrant mucin O-glycans. Despite its biological relevance, there is a lack of knowledge regarding the conformational behavior of STn derived structures in solution. Likewise, the conformational studies of ligands containing sialic acid α2,6-linked to an internal GlcNAc, typically found in milk derivatives (e.g. sialyllacto-*N*-tetraose b), are absent and should be investigated.

### Conformations Adopted by α2-8 Sialoglycans

The α2-8 linkage is mostly present in polysialic acid (PolySia) oligomers at the terminal residues of higher gangliosides, such as disialo- (GD), trisialo- (GT), and tetrasialo- (GQ) gangliosides, and as the terminal motif of specific glycoproteins (Vasudevan and Balaji, 2002; Varki et al., 2015). This linkage is defined by five torsion angles, φ (C1-C2-O-C8′), ψ (C2-O-C8′-C7′), ω9 (O9′-C9′-C8′-O), ω8 (O8′-C8′-C7′-O7′), and ω7 (O7′-C7′-C6′-O6′), which offer significant conformational flexibility and increase the distance between the pyranose rings in the sialoglycan, originating a large conformational space distribution (Vasudevan and Balaji, 2002; Turupcu et al., 2020).

**FIGURE 2:**
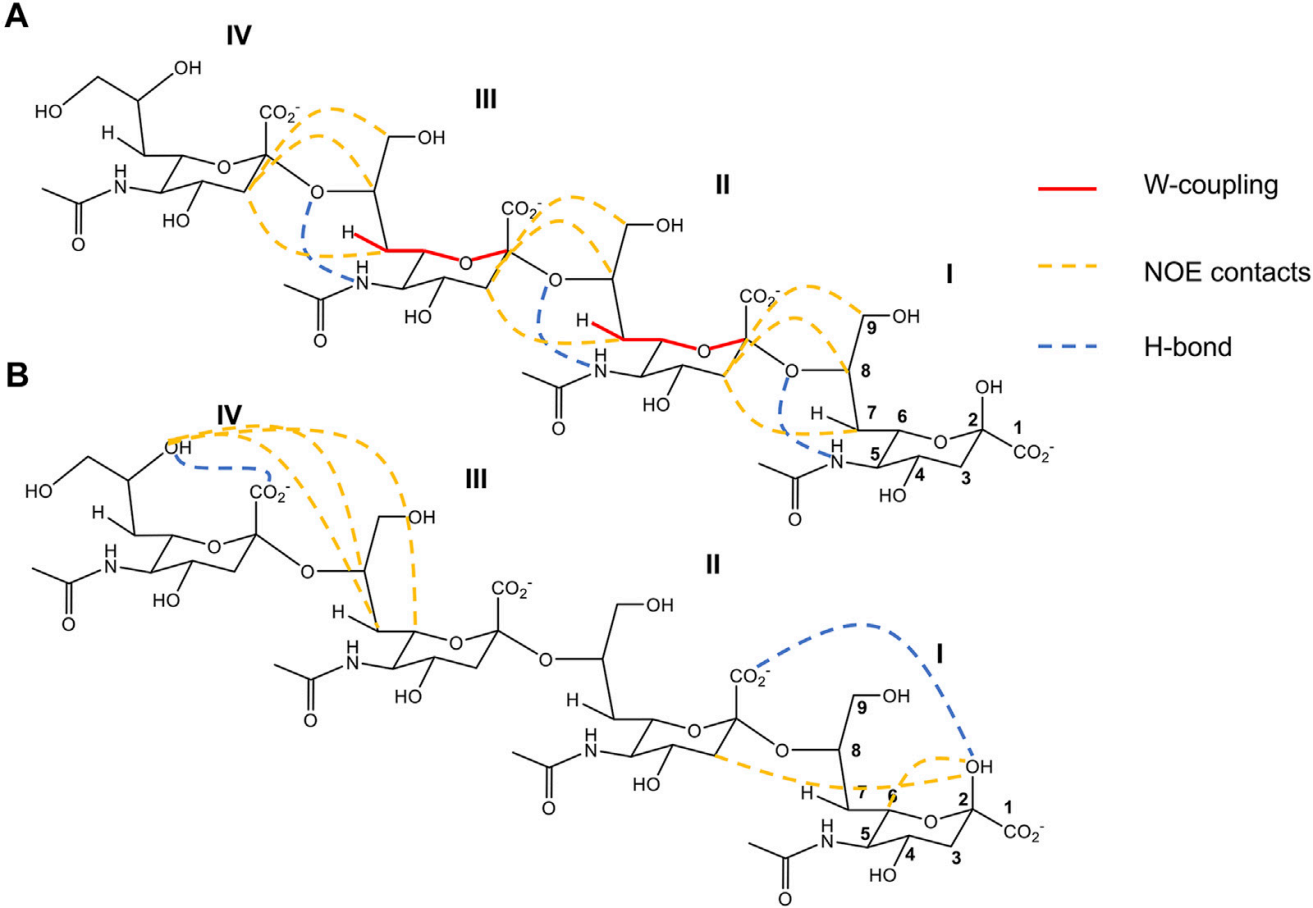
2-D structure representation of (Sia)_4_ with the most important structural features deduced by Battistel et al., 2012 (A) and Novakovic et al., 2021 (B). **(A)**. Contacts compatible with the left-handed helix conformation obtained in a supercooled aqueous solution (263 K). In red lines are represented the W-coupling between C2 and H7, in yellow dashed lines the principal distances derived from NOEs contacts, and in blue dashed line the intra-residue H-bond deduced between O8 and NH5. **(B)**. Contacts compatible with a model combining rigid and flexible segments obtained in closer physiological conditions. In yellow dashed lines are represented the principal distances derived from NOE contacts. In blue dashed lines are the H-bonds IIO1-IOH2, consistent with a rigid conformation in the reducing end of (Sia)_4_, and IVOH8-IVO1 the principal structural motif for the most flexible part of the nonreducing end. Figures adapted from Battistel et al. (2012) and Novakovic et al. (2021).

PolySia are linear α2-8 linked homopolymers of Neu5Ac with a degree of polymerization varying from 8 to 200 Neu5Ac units (Sato and Kitajima, 2008). Antibodies recognize PolySia polymers with 10 or more Neu5Ac residues as minimal epitope (Jennings et al., 1985; Turupcu et al., 2020). Therefore, it was hypothesized that PolySia structures with a certain degree of polymerization adopt a specific conformation in solution that would promote the binding of antibodies (Azurmendi et al., 2017). In this regard, several conformational studies were carried out on PolySia with different degrees of polymerization (Henderson et al., 2003; Battistel et al., 2012; Hanashima et al., 2013; Turupcu et al., 2020; Novakovic et al., 2021). However, no consensus is found in the literature regarding the PolySia conformation, which can alternate between random coil and distinct types of helical conformations. It was suggested in 2012, based on heteronuclear J couplings and the inter-residue NOEs of a ^15^N,^13^C tetramer of Neu5Ac (Sia)_4_ in a supercooled aqueous solution (263 K), about the existence of an H-bond that would support a helix-like conformation in PolySia structures (Battistel et al., 2012). Based on the long range coupling constant analysis (CBCANH and HNC2) and ^1^H/^2^H exchange rates (SOLEXSY) was postulated the existence of an intra-residue H-bond between the HN and O8 of the Neu5Ac I-III residues (Sia I-III) (Figure 2A). This H-bond is translated into a restricted flexibility of the glycerol side chain, which was further experimentally supported by the heteronuclear coupling constant between H7 and C2. This coupling indicates that the H7-C7′-C6′-H6 torsion angle is ∼90° and that H7 adopts a coplanar or quasi-coplanar W conformation with C2. Static models were created for the tetramer applying the NMR results as restraints, yielding two possible structural models: a left-handed helix with 2 or a helix with 4 residues/turn. Finally, intra- and inter-residue NOEs suggested that (Sia)_4_ adopts a left-handed-like helical conformation. NOEs between H7, H8, and H9 of a Neu5Ac residue and both H3_ax_/H3_eq_ of the following Neu5Ac residue for Sia I-III of (Sia)_4_ were detected. These NOEs are consistent with the intra-residue H-bond between HN and O8, and compatible with a left-handed helix model. The nonreducing end in the tetramer (Sia IV) seems to behave as a free sialic acid unit. However, the structure derived from the combination between the NMR and computational models is not consistent with the exo anomeric effect (Battistel et al., 2012; Turupcu et al., 2020). In 2020, the molecular dynamic simulations of di-, tri-, tetra-, and deca-α2-8-linked sialic acid structures were carried out to study PolySia conformational preferences in solution (Turupcu et al., 2020). In these simulations, several inter-residue H-bonds between the successive residues were observed. However, the intra-residue H-bond between HN and O8 was rarely observed. In the PolySia decasialic acid structure simulations, only 37% of the structures showed helical patterns, which supports a flexible conformation for PolySia oligomers and excludes the left-hand helix model suggested by Battistel et al. in 2012 (Turupcu et al., 2020). Therefore, Turupcu et al. proposed that even though helical conformations were unlikely to play a dominant role in free PolySia structures, they could be induced upon binding. Additionally, Hanashima and collaborators found differences in the inter-residue NOE correlations between the bound and free conformations of PolySia polymers (Hanashima et al., 2013). The authors concluded that PolySia structures hold multiple conformations in solution; however, a minor and unfavorable conformation can be adopted upon binding to an antibody or within a glycoprotein environment.

Recently, high-field Looped-PROjected spectroscopy (L-PROSY) NMR was employed to revisit the (Sia)_4_ conformation closer to physiological conditions in a solution at 278 K (Novakovic et al., 2021). This experiment enhances the NOESY and TOCSY cross-peaks for ^1^Hs that undergo fast exchange with water, and it is ideal to extract structural information from hydroxyl groups in sugars. The authors observed an H-bond between OH_2_ of Sia I and O_1_ of Sia II, which dictates a well-defined conformation on the reducing end residues of (Sia)_4_ (Figure 2B). This structural motif was previously observed in shorter (Sia)_2_ homologue, and it was suggested to be a common feature of PolySia structures (Azurmendi et al., 2017). For the nonreducing end (Sia III-IV residues), the authors reported high potential flexibility together with the typical structural motif of the glycerol side chain of the terminal end Sia IV, characterized by the stable intra-residue H-bond between OH_8_ and O1 (Novakovic et al., 2021). Notably, a direct through-hydrogen-bond correlation was detected via long-range HSQMBC between OH_8_ and C1, compatible with a stable H-bond between OH8 and O1 of Sia IV (Figure 2B). Thus, Novakovic et al. proposed a structural model for (Sia)_4_ in solution that combines rigid motifs with flexible segments. However, the influence of a longer PolySia oligosaccharide chain on these structural features is still unknown and needs further investigation.

Understanding the structural aspects of sialic acid-containing glycoconjugates in solution is relevant to understand their biological functions and for the rational design of sialic-based mimetics. However, conformational studies at a free state are insufficient, since a ligand when complexed with a receptor tends to adopt different conformations and dynamic properties. In this perspective, in the following sections of this review, we will discuss how sialoglycan’s conformation is modified upon binding with different receptors.

## Sialic Acid—Siglec Interactions

The sialic acid-binding immunoglobulin-like lectins (siglecs) are a family of cell surface receptors mainly expressed in the immune system cells, which specifically recognize sialic acids and modulate immune responses through cell signaling (Crocker et al., 2007; Duan and Paulson, 2020). They are classified as I-type lectins, since they present a variable number of extracellular domains homologous to the immunoglobulin (Ig) superfamily of proteins (Crocker et al., 2007). These include the carbohydrate recognition domain (CRD) at the N-terminal, which has a shallow binding pocket comprising an essential Arg residue that establishes a key salt bridge with the carboxylate group of the sialic acid (Crocker et al., 2007; Duan and Paulson, 2020; Movsisyan and Macauley, 2020). As the sialic acid is naturally present at the terminals of human glycans and in pathogens, their recognition by siglecs is an important process in distinguishing the self from the non-self in humans (Duan and Paulson, 2020; Läubli and Varki, 2020). In addition, the sialylation pattern and density also change in certain diseases, namely in cancer, which can be also attended with the alteration in the expression of some siglecs. In this context, the study of siglecs and their interactions with sialylated ligands becomes an attractive strategy to develop therapies targeting siglecs (Angata et al., 2015; Duan and Paulson, 2020; Läubli et al., 2020; Läubli and Varki, 2020).

There are fourteen siglecs in humans, but only seven (sialoadhesin, CD22, CD33, MAG, Siglec-5, Siglec-7, and Siglec-8) have their structures reported (of the CRD domain, at least), either by X-ray crystallography or NMR (May et al., 1998; Alphey et al., 2003; Attrill et al., 2006; Zhuravleva et al., 2008; Macauley et al., 2014; Pronker et al., 2016; Pröpster et al., 2016; Ereño-Orbea et al., 2017; Miles et al., 2019; Duan and Paulson, 2020; Movsisyan and Macauley, 2020). Additional NMR studies assisted with molecular modeling have been undertaken to uncover siglec interactions with sialoglycans even when the 3-D structure of the lectin is not known (Bhunia et al., 2004; Barb et al., 2013; Madge et al., 2016; Pröpster et al., 2016; Forgione et al., 2020a; Di Carluccio et al., 2020; Yamakawa et al., 2020). This approach has proven robust and versatile to study the molecular interactions of sialic-containing ligands and siglecs.

A simple early example, which demonstrates the validity and usefulness of combining saturation transfer difference (STD) NMR with molecular modeling in the study of siglecs, is the characterization of the binding epitope of 3′-sialyllactose (Neu5Acα2-3Galβ1-4Glc, 3′SL) when interacting with sialoadhesin (Siglec-1) (Bhunia et al., 2004). Through the STD–NMR data, the authors identified that the major region of the ligand in contact with the binding site of sialoadhesin is the sialic acid, reporting the largest STD effect for the *N-*acetyl methyl group of the Neu5Ac unit, which indicates that this group is oriented toward the binding pocket. A slight contribution of the galactose unit for the binding event was also noticed, while the glucose did not receive any saturation. Simultaneously, a molecular docking model of the complex was generated using a crystal structure of a sialoadhesin complexed with 3′SL (PDB ID: 1QFO) to predict the theoretical STD enhancements through CORCEMA–STD analysis. A good agreement between the experimental and theoretical STD values illustrates that the combined modeling/STD NMR approach is a valuable methodology to provide a reliable structural model for sialoadhesin/3′SL. The model evidences a great number of interactions between the sialic acid and the protein, highlighting the hydrophobic interaction between the *N-*acetyl methyl group and the aromatic ring of the Trp2 in the protein. The galactose residue only performs two minimal contacts with the binding pocket.

A recent work also uses ligand-based NMR approaches combined with computational methods to understand the binding events led by siglec-10 (Forgione et al., 2020a). STD–NMR and tr-NOESY experiments with 3′SLN and 6′SLN were carried out to obtain STD-derived epitope maps and bioactive conformations, respectively, in the presence of siglec-10. The STD–NMR results expectedly showed that, for both ligands, the key residue for the interaction is the Neu5Ac unit. In both 3′SLN and 6′SLN, the highest STD response corresponds to the *N*-acetyl methyl group, followed by strong STD effects on the H6 and H7 of the Neu5Ac residue. However, some differences between ligands were observed: for 3′SLN, other protons of Neu5Ac receive significant saturation from the protein, contrarily to that observed for 6′SLN. Additionally, the Gal unit of 3′SLN seems to be more involved in the interaction with siglec-10 than that of 6′SLN. In the case of 3′SLN, tr-NOESY indicates a conformational selection of the -g conformer (φ = −60°) upon binding (3′SLN has two stable conformers in solution, the -g and t conformer), while for 6′SLN, the data suggest that the gt geometry (φ/ψ/ω of −60°/180°/60°) around the Neu5Acα2-6Gal linkage is the most populated in both free and bound states. Tridimensional views of siglec-10/3′SLN and siglec-10/6′SLN with the ligands adopting different geometries were derived from docking calculations and molecular dynamics (MD) simulations using a homology model of siglec-10. The theoretical STD responses were further predicted using the CORCEMA-ST protocol, which confirmed the bioactive conformations suggested by tr-NOESY for 3′SLN and 6′SLN to siglec-10. The models revealed that the binding site of siglec-10 is superficial (as it happens with its family members), and the sialic acid is the main binding determinant performing several polar and hydrophobic interactions with the protein surface. In contrast, Gal is less involved and GlcNAc is pointing outward from the lectin. The authors also studied the interaction of siglec-10 with longer naturally occurring biantennary *N-*glycans, with both types of linkages at the extremities, and obtained comparable results to those of small ligands, validating the same conclusions for more complex sialoglycans.

Similar NMR and molecular modeling approaches were also used to investigate the binding of complex sialylated *N*-glycans to CD22 (Siglec-2) (Di Carluccio et al., 2020). For comparison purposes, the authors first studied the interaction of CD22 with 6′SLN. Unsurprisingly, the contacts with the protein are made mainly through the sialic acid residue, with the Gal unit helping to stabilize the orientation of the ligand into the binding site and where the GlcNAc moiety reveals a dynamic behavior. This observation agrees with the co-crystal structure of CD22 with α2-6-sialyllactose (6′SL) (PDB ID: 5VKM) (Ereño-Orbea et al., 2017). To evaluate the response with different biantennary glycans, the authors used STD–TOCSY experiments along with MD simulations. CD22 recognizes only the terminal disaccharide portions of the *N*-glycan, regardless of the branch to which they are attached, and reveals that the binding mode to CD22 is superficial and not influenced by the internal sugar residues of the *N*-glycan. The interaction of Neu5Gc derived ligands with CD22 was also investigated (Di Carluccio et al., 2021) and demonstrated that the Neu5Gc ligand exhibits a similar binding epitope and orientation to CD22 than the Neu5Ac counterpart.

On-cell STD–NMR was also applied to monitor interactions between the CD22 present on living Burkitt’s lymphoma (BL) Daudi cells and the sialic acid based mimetics (Madge et al., 2016). As a starting structure, the authors used a sialic acid mimetic substituted at C4 and C9, and after several STD controls, they confirmed that the STD responses were only detected in cells that express CD22. The STD-derived epitope map of the first mimetic allowed the authors to rationally design further modifications at C2 and C3 of the sialic acid moiety. The new ligands, in turn, were evaluated and showed higher affinities than the first mimetic. This work proves the usefulness and effectiveness of on-cell STD–NMR methodologies in a structure-guided approach for the development of glycomimetics targeting siglecs under more biologically relevant conditions.

Molecular modeling and NMR were also useful in an interesting discovery of a secondary sialic acid binding site, working as a regulatory site, in siglec-7 (Yamakawa et al., 2020). This hypothesis came up after a docking analysis using different X-ray derived structures of siglec-7 and a disialylated glycan, diSiaGal (Neu5Acα2-8Neu5Acα2-3Gal-Me), which revealed a lower binding energy complex in an unforeseen location (involving R67 at the C-C′ loop instead of the conventional R124 located at the F strand of siglec-7). To support this finding, STD–NMR experiments of a di- and trisaccharide of α2,8-linked Neu5Ac units in the presence of siglec-7 wildtype (WT) and two mutated variants (R124A and R67A) were performed. In the presence of siglec-7 WT and the mutant R67A, both ligands are recognized mainly through the nonreducing terminal residue of PolySia structures. In the presence of the mutant R124A, the STD response reduces and the preference toward the nonreducing unit of the di- and tri-sialic derivatives decreases. Thus, the authors concluded that the mutation at R124 precludes binding at the primary sialic acid site of siglec-7 located at the F strand; however, it also induces a conformational change in the C-C’ loop able to modulate the binding of PolySia structures (involving R67) at the secondary binding site.

**FIGURE 3:**
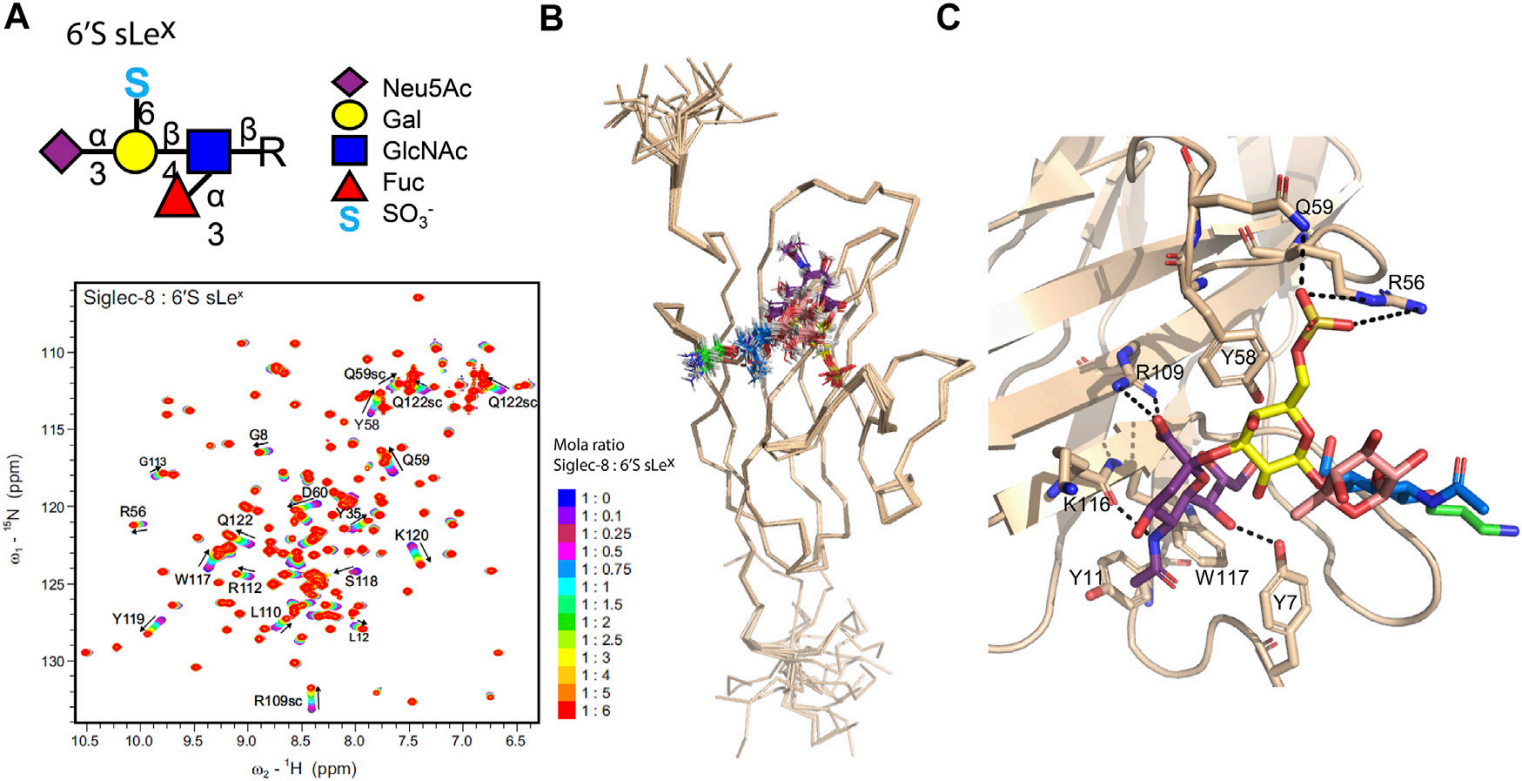
Human siglec-8/6′S sLe^x^ complex deduced by the NMR and molecular modeling. **(A)**. ^1^H,^15^N-HSQC based titration of siglec-8 with increasing amounts of 6′S sLe^x^. The successive ^1^H,^15^N spectra of siglec-8 collected after the gradual addition of 6′S sLe^x^ are superimposed. The spectrum of siglec-8 in the absence of 6′S sLex is displayed in dark blue, and the spectrum of siglec-8 in the presence of 6 equivalents of 6′S sLe^x^ is displayed in red. The most perturbed residues are identified, and the directions of the peak’s shifts are indicated with arrows. **(B)**. An ensemble of the 20 structures with the lowest energy of siglec-8 complexed with 6′S sLe^x^ determined by NMR. **(C)**. Representation of the siglec-8/6′S sLe^x^ interface, where the 6′S sLe^x^ and the interacting amino acids are displayed as sticks and the hydrogen bonds between Neu5Ac/Gal 6′-sulfated and siglec-8 are illustrated as dashed lines. Adapted from Pröpster et al. (2016).

Protein-perspective NMR approaches were also applied in the binding studies of siglec-5 (Barb et al., 2013) and siglec-8 (Pröpster et al., 2016). ^1^H,^15^N-HSQC titration experiments of the siglec CRDs with increasing amounts of sialic-containing ligands were performed to identify the ligand binding site. Gradual changes in the chemical shift of the signals in the spectra of both studies reveal a fast exchange regime between the free and bound states of the lectins, and K_D_ values consisted of a weak binding in agreement with siglec’s mechanism of recognition for small sialic-containing fragments (only low values of K_D_ were observed in the case of sulfated sLeX derivatives). Regarding siglec-5 (Barb et al., 2013), the monosaccharide Neu5Ac and the 3′SLN and 6′SL derivatives were evaluated. Despite their structural differences, the three ligands bind at the same recognition site; however, in the case of 3′SLN and 6′SL, the extent of the perturbation is greater than that of the monosaccharide due to the presence of the two additional sugar moieties. The lower K_D_ values for 3′SLN and 6′SL reveal that the presence of Galβ1-4GlcNAc in 3′SLN and Galβ1-4Glc in 6′SL enhances the affinity toward siglec-5. A more exhaustive structural study of siglec-8 (Pröpster et al., 2016) allowed evaluation of the fine specificity of sulfated Neu5Ac derivatives. The authors tested a group of different sLe^x^ glycan derivatives (Gal- and GlcNAc 6-sulfated sLe^x^ and corresponding nonsulfated sLe^x^), in which all displayed similar chemical shift perturbations (CSP) (Figure 3A), indicating an identical binding site. However, the K_D_ analysis indicates that siglec-8 has a higher affinity toward the Gal-6-sulfated sLe^x^ glycan epitope (6′S sLe^x^) than to GlcNAc-6-sulfated sLe^x^ or nonsulfated sLe^x^ analogues. The 3-D structure of siglec-8 complexed with 6′S sLe^x^ was also deduced by NMR, which showed the existence of intermolecular NOEs between 6′S sLe^x^ and the specific amino acids of the siglec-8 binding site (Figure 3B). The Neu5Ac residue plays an essential role, participating in several non-covalent interactions, similar to those observed in other siglecs, while the fine specificity is given by the recognition of the sulfated Gal through interactions with a unique loop of siglec-8 (Figure 3C).

In summary, siglecs are an extensive family of lectins where all their members recognize the same primary epitope, the sialic acid motif, leading to a general specificity toward sialic acid glycoconjugates. The salt bridge involving the Neu5Ac carboxylate group and the conserved arginine residue of siglec’s family is the key contact point for the recognition process. However, this recognition is improved through additional interactions between the sialic acid structure and the characteristic siglec’s solvent-exposed binding surface. The *N*-acetyl or *N*-glycolyl group of Neu5Ac and Neu5Gc, respectively, strongly contributes to the binding event, being engaged in hydrophobic interactions. The adjacent sugar residues of sialic acid are less involved in the recognition. However, the type of linkage that connects sialic acid to other sugars, the type of other sugars that make up the glycan (and their chemical modifications), and the conformation that the sialoglycoside acquires upon binding seem to underlie the fine specificity between siglec’s members. From this perspective, it is relevant to discover the molecular determinants from the protein and sialoglycan viewpoint that trigger the fine specificity concerning more complex and functionalized sialoglycans. This structural information can be extracted from the NMR in combination with molecular modeling and is of utmost importance for the design of selective drugs for each siglec member.

## Sialic Acid—Virus Interactions

The process by which viruses infect cells starts with their attachment to the host cell surface using stalk-like proteins, the adhesins or agglutinins, which bind to cell surface receptors, namely, proteins or glycans in glycoproteins or gangliosides, including the sialylated ones (Stencel–Baerenwald et al., 2014; Matrosovich et al., 2015). The specificity of the distinct viral proteins regarding the sialoglycans influences the viral tropism, pathogenicity, and transmissibility (Matrosovich et al., 2015). For that reason, the virus’s lectins (adhesins) and the interactions they establish with the host sialoglycans are an interesting target to understand viral infections and develop therapies, such as drugs and vaccines, and also viral vectors (Stencel–Baerenwald et al., 2014). Some examples of sialic acid-binding viruses whose interactions with the host sialoglycans have been studied by NMR with the assistance of molecular modeling protocols will be reviewed herein, namely, concerning the rotavirus (Haselhorst et al., 2011), the influenza virus (Fernández de Toro et al., 2018; Vasile et al., 2018b, 2018a), the adenovirus (Lenman et al., 2018), and the polyomavirus (Neu et al., 2013; Ströh et al., 2020) classes.

Rotaviruses adhere to the intestinal cell glycan receptors through the trimeric virion spike protein VP4, whose subunit VP8* is responsible for the binding event (Haselhorst et al., 2009; Stencel–Baerenwald et al., 2014). The interaction of the recombinantly expressed VP8* from two strains with branched ganglioside GD1a and GM1 fragments was first studied by STD–NMR, which revealed the relevance of the Neu5Ac moiety in the interaction between the virus and the host cell (Haselhorst et al., 2009). Later, the interaction of the GM3 glycan (Neu5Acα2-3Galβ1-4Glc, 3′SL) with VP8* present on rhesus rotavirus (RRV) particles was also investigated by STD–NMR and tr-NOESY experiments (Haselhorst et al., 2011). The highest STD enhancements arise from the methyl group of the *N-*acetyl moiety and the diastereotopic H3_ax_/H3_eq_ protons of Neu5Ac. A significant contribution of the Gal unit was also noticed for 3′SL, unlike to that observed for α2,6-linked counterparts. Through tr-NOESY analysis and molecular modeling, the bioactive conformation of GM3 to VP8* from RRV was deduced. The disappearance of the interglycosidic NOE crosspeak between H3_ax_ Neu5Ac and H3 Gal in the bound state (characteristic of t conformer) indicates that the VP8* selects the -g conformer of the Neu5Acα2-3Gal portion as the bioactive conformer.

Influenza viruses are important respiratory pathogens that affect humans and animals. They have two important viral proteins at the surface, the hemagglutinin (HA) and the neuraminidase (NA), both interacting with sialic acid receptors present in the host (Matrosovich et al., 2015). Focusing on the HA, it is a trimeric protein essential for the viral attachment and consequent fusion of the viral and cell membranes (Matrosovich et al., 2015). Recently, the binding of 3′SLN and 6′SLN to H5 and H1 strains (avian and human HAs, respectively) expressed on 293 T cells was investigated by STD–NMR and trNOESY based experiments (Vasile et al., 2018b). The STD–NMR results show that both 3′SLN and 6′SLN interact with H1 and H5 strains, however through different binding modes. In the case of 3′SLN, the main determinant of the interactions is the Neu5Ac residue, with the emphasis laid on the *N*-acetyl group. In contrast, for 6′SLN, all three residues seem to be involved in the H1 and H5 recognition. The STD-derived epitopes perfectly correlate with the glycan bioactive conformations, which were deduced by the qualitative analysis of the tr-NOESY spectra. Thus, 3′SLN exhibits an extended bioactive conformation, limiting the contact with the protein to the nonreducing terminal sugar, whereas 6′SLN adopts a bent conformation, allowing the contact of the three sugar residues with the binding site. From the STD data, the authors also concluded that 3′SLN is similarly recognized by H1 and H5, while the binding of 6′SLN is stronger for H5 than for H1. This study highlights that on-cell NMR ligand-based methods are robust and useful to characterize sialoglycan binding to the proteins present on transfected human cells, thereby allowing unraveling of the molecular determinants of the recognition event in a physiological-like environment, thus avoiding the recombinant expression of proteins or the use of virus-like particles (Vasile et al., 2018a).

**FIGURE 4:**
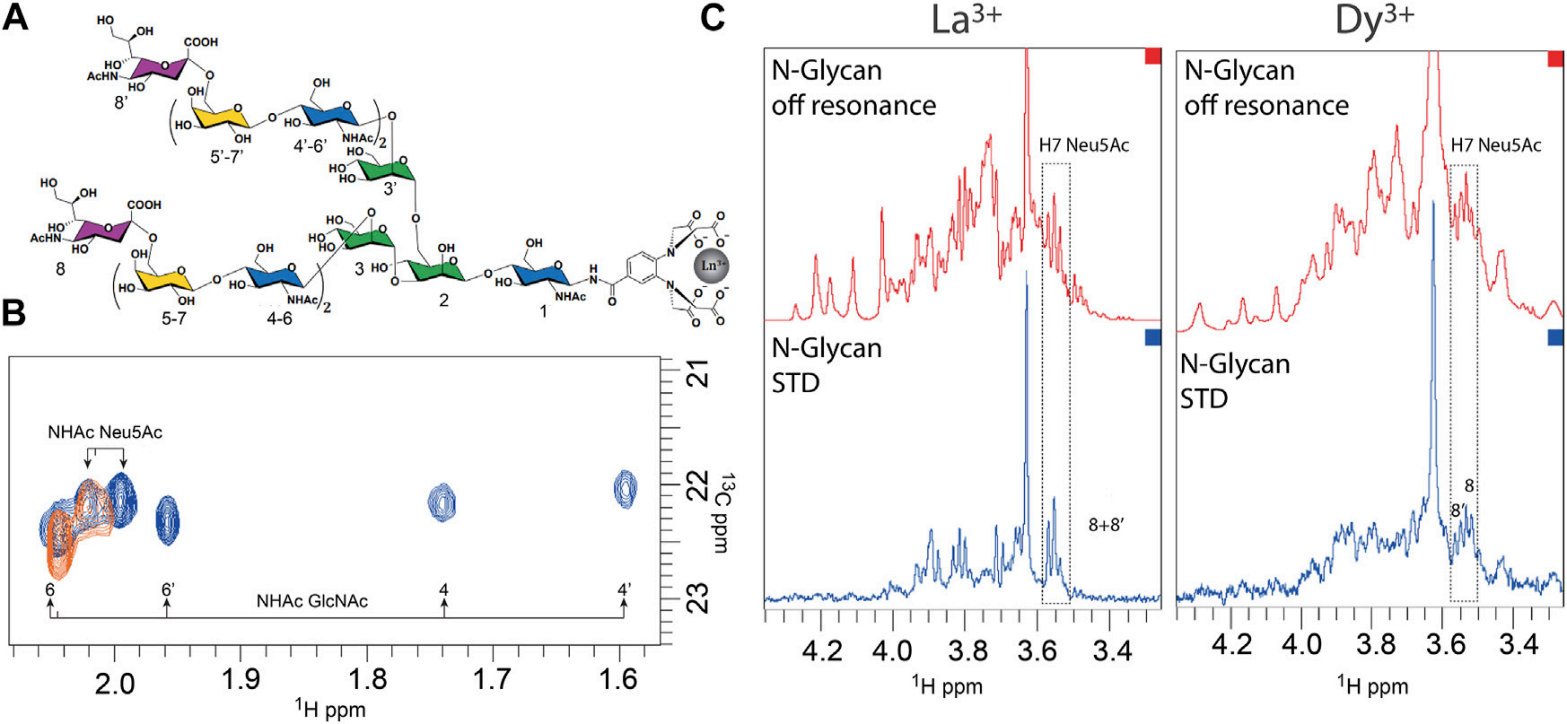
Recognition of a complex *N*-glycan by the hemagglutinin from HK/68 H3N2 influenza virus deciphered by NMR (Fernández de Toro et al., 2018). **(A)**. Structure of the *N*-glycan with the corresponding numbering of the 14 monosaccharides. **(B)**. Superimposition of ^1^H–^13^C HSQC spectra in the region of the NHAc methyl group of GlcNAc and Neu5Ac of the *N*-glycan loaded with La^3+^ (diamagnetic, in orange) and with Dy^3+^ (paramagnetic, in blue). **(C)**. STD–NMR experiment of *N*-glycan in the presence of La^3+^ (diamagnetic conditions, left) and loaded with Dy^3+^ (paramagnetic conditions, right). Adapted from Canales et al. (2017).

An innovative NMR strategy (Canales et al., 2017) was employed to study the interaction of a complex *N*-glycan with the hemagglutinin from A/HongKong/1/1968 (HK/68) H3N2 influenza virus (Fernández de Toro et al., 2018). This strategy uses paramagnetic NMR to resolve and distinguish the branches of a complex *N*-glycan (Figure 4A), which in normal conditions would have overlapping resonances due to the symmetry of the branches and the repetition of monosaccharides under similar chemical environments. To enable this approach, a lanthanide-binding tag (LBT) must be linked to the reducing end of the glycan. The LBT produces pseudo-contact shifts (PCSs) depending on the distance and orientation between the lanthanide and the protons. Since each arm has a unique geometry in relation to the metal, they experience different PCS values (the closer the proton is to the LBT tag, the larger will be the PCS), which allow discriminating each arm of the *N*-glycan (Figure 4B). These values can be further translated into distances and serve as inputs to calculate conformational geometries in combination with molecular dynamics simulations. In the study with the HA, the authors were able to analyze the interaction of a bi-antennary *N-*glycan with 14 monosaccharides, comprising two *N*-acetyllactosamine (LN) repeats, and with an α2,6-linked Neu5Ac unit at the terminal of each branch. Unlike the chemical shift degeneration that is observed under diamagnetic lanthanum conditions (La^3+^), in the presence of the paramagnetic dysprosium (Dy^3+^), the proton signals are isolated and allow the unambiguous identification of every sugar residue’s signals. Hence, further STD–NMR studies were used, which demonstrate that the HA interacts independently with each Neu5Ac at both arms of the glycan (Figure 4C).

This work illustrates that the combination of a paramagnetic tag, strategically located in the ligand, with the traditional ligand-based NMR binding techniques offers the possibility to investigate more and more complex glycans.

Most of the adenoviruses use protein receptors, but some serotypes specifically interact with sialylated glycan receptors, employing a trimeric fiber protein with a terminal globular structure (knob) as a mediator of the viral attachment (Stencel–Baerenwald et al., 2014). The human adenovirus 52 (hAdV-52) is one of the few types of adenoviruses with two kinds of fiber proteins, designated as long and short. The short fiber knob (52SFK) has been shown to recognize long α2,8-linked PolySia structures commonly present in some carrier proteins (Lenman et al., 2018). The interaction preferences of 52SFK were investigated through the integration of distinct techniques: glycan microarrays, STD-NMR, structure-guided mutagenesis, X-ray crystallography, and MD simulations. The data determined the preference of the 52SFK toward PolySia structures containing multiple α2,8-type linkages rather than 2,3- and 2,6-linked sialic acids. In addition, the maximal affinity was observed for a degree of polymerization higher than 3 (DP5–9). The STD–NMR spectra of PolySia (DP3 and 5) show that the transfer of magnetization occurred mainly due to the nonreducing end unit, whereas the other residues only receive moderate saturation on the *N*-acetyl group. This result agrees with the crystal structures of 52SFK complexed with PolySia (DP3, 4, and 5) that show a very well-defined electron density for the Neu5Ac at the nonreducing end and only a weak electron density for the second Neu5Ac residue. STD–NMR experiments also indicate that PolySia oligomers only interact in the canonical sialic acid binding site of 52SFK since no STD response was observed in the presence of the mutant R316A 52SFK. Finally, MD simulations of the 52SFK/PolySia (DP5) complex were accomplished, confirming that the nonreducing Neu5Ac is mandatory for the binding and thereby suggesting that the fine specificity is provided through transient electrostatic interactions between the 52SFK and the additional sialic acid units.

Most polyomaviruses (PyVs) use the pentameric VP1 protein to recognize and attach to sialylated glycans at gangliosides or glycoproteins from the host cell membrane (Blaum et al., 2018). The characterization of the specificity of some human PyVs has been done in an integrative fashion combining several techniques (Neu et al., 2013; Ströh et al., 2020). For the human polyomavirus BKPyV (Neu et al., 2013), the STD–NMR experiments of GD3 and GD1b glycans in the presence of VP1 reveal a common epitope, the α2,8-linked disialic acid portion of the glycans, where the *N*-acetyl groups are the main receivers of saturation. However, if for GD3 the Gal-Glc moiety does not show an STD response for the left arm of GD1b, consisting of a Gal and a GalNAc residue, an additional transfer of saturation is observed, which indicates extra contacts with VP1. The STD-derived epitope of GD3 agrees with the X-ray crystallography structure obtained for the VP1/GD3 complex (PDB ID: 4MJ0) that highlights relevant contacts with the disialic motif and the absence of interactions for the remaining sugar units. Additionally, the complex VP1/GD1b derived from molecular modeling using the VP1/GD3 structure as a starting model corroborates the STD data, exhibiting several weak interactions between the left arm of GD1b and the protein. Interestingly, the STD–NMR data also demonstrated that the mutation of a single residue in the binding site of BKPyV VP1 alters its specificity, precluding the binding to GD3 but enabling the binding to GM1. The specificity of VP1 proteins from the polyomaviruses NJPyV and HPyV12 were also investigated by STD–NMR in combination with the X-ray crystallography (Ströh et al., 2020). While HPyV12 VP1 interacts with both 3′SL and 6′SL, NJPyV VP1 specifically binds to 3′SL, but it is not able to recognize 6′SL. In the case of HPyV12 VP1, the Neu5Ac unit common to both 3′SL and 6′SL ligands is the only unit displaying STD enhancements, which suggests that Gal-Glc portion is solvent-exposed and does not perform contacts with the protein. These observations agree with the X-ray structure deduced for the HPyV12 VP1/3′SLN complex (PDB ID: 6Y60) and might indicate that the binding site of the virus could accommodate α-2,3 and α-2,6-linked Neu5Ac-containing ligands. In the case of NJPyV VP1, only the 3′SL shows an STD response and no resonances of 6′SL were detected in the STD spectrum, suggesting the relevance of the α-2,3 Neu5Ac-Gal glycosidic linkage in the interaction with the protein. Nevertheless, for 3′SL only, Neu5Ac moiety receives saturation from the protein and no resonances of the Gal-Glc part were detected in the STD spectrum. The importance of the α-2-3 glycosidic linkage in the interaction was further supported by the lack of the STD response of 2-*O*-methyl-α-Neu5Ac (isolated sialic acid) in the presence of NJPyV VP1. This result is also consistent with the X-ray crystallography structure of the NJPyV VP1/3′SL complex (PDB ID: 6Y5Y), which demonstrated that the Gal unit performs a smaller number of interactions than Neu5Ac, however essential for ligand recognition.

In brief, a common structural signature of sialic acid recognition by viruses is the interaction of the NHAc group of Neu5Ac into a hydrophobic pocket of the viral protein. Nevertheless, the fine specificity of viruses toward sialic acid glycans is dictated by the type of glycosidic linkage to which Neu5Ac is attached (α-2,3, α-2,6-, or α-2,8-linkage) and by the composition and the conformation of the sialoglycan, in combination with the architecture of the viral protein binding site. Hence, sialoglycans can exhibit distinct binding epitopes and bioactive conformations when interacting with viral proteins. Interestingly, single point mutations in viral proteins can shift the preference of the viruses toward the sialoglycan. In this perspective, the NMR in combination with X-ray data has contributed to identify and describe the structural details in sialic acid glycan recognition mechanism for several viral proteins. This structural information is of utmost importance to understand viruses’ specificity, infection, and tropism as well in the rational design of multivalent glycomimetics to block the sialoglycan/viral adhesin interactions.

## Sialic Acid—Bacteria Interactions

The constant communication between the host and the bacteria contributes to 1) the tolerance of the bacterial species inside the host that leads to listless host habitation and/or 2) the resistance and replication of the bacterial species with damage for host cells that leads to diseases. Generally, the first contact between the bacteria and the host cells involves carbohydrate–protein interactions, which can occur in distinct ways: the host receptors recognize glycans present on bacteria, bacterial adhesins bind to the host glycans present on the host-cell surface, and/or an interesting characteristic of pathogen invasion and colonization, which consists in the use of glycosidases or glycosyltransferases to modify the host glycans, in order to improve their adhesion capacity and biofilm formation or to acquire the potential nutrient source (Martens et al., 2008; Tailford et al., 2015a; Pudlo and Martens, 2015). Sialic acids (Neu5Ac) and fucose residues are generally present in terminal positions on the host glycan chains and are prominent targets for commensal and pathogenic bacteria (Park et al., 2016, Park et al., 2017). In this context, several bacteria use sialidases to promote bacterial survival in mucosal environments. There are two types of sialidases: the hydrolytic sialidases that cleave terminal sialic acids and release free Neu5Ac and *trans*-sialidases that transfer the cleaved sialic acid to other glycoconjugates.

**FIGURE 5:**
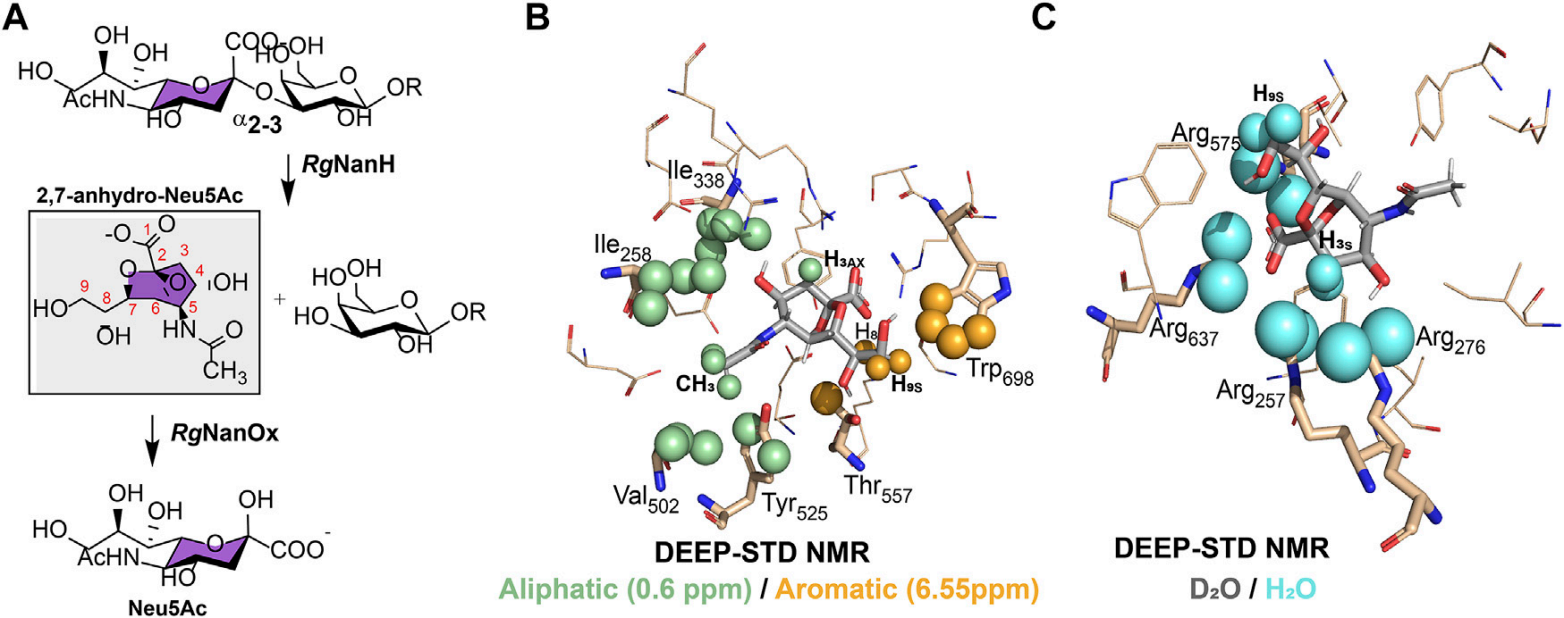
Metabolism and recognition of sialic acid structures by *Ruminococcus gnavus* ATCC 29149 strain. **(A)**. Schematic representation of the enzymatic processing of α2,3-linked sialic acid compounds to 2,7-anhydro-Neu5Ac by *Rg*NanH and reversible conversion of 2,7-anhydro-Neu5Ac to Neu5Ac by *Rg*NanOx. **(B)** and **(C)**. Differential epitope mapping of 2,7-anhydro-Neu5Ac in complex with *Rg*NanH-GH33. The crystal structure of the complex (PDB ID: 4 × 4A) (Tailford et al., 2015b). **(B)**. DEEP–STD map (0.60/6.55 p.p.m) of 2,7-anhydro-Neu5Ac. Green spheres highlight ligand contacts with aliphatic side chains, and orange spheres represent contacts with aromatic side chains. **(C)**. DEEP–STD map (D_2_O/H_2_O) of 2,7-anhydro-Neu5Ac. Cyan spheres show ligand contacts with protein side chains carrying slow exchanging protons. Adapted from Monaco et al. (2017).

*Ruminococcus gnavus* (*R. gnavus*) is a commensal anaerobic Gram-positive bacterium, which has been part of normal intestinal flora in humans. *R. gnavus* (*Rg*) ATCC 29149 strain expresses a sialidase, *Rg*NanH, characterized by a catalytic domain (*Rg*NanH-GH33), responsible for the hydrolysis, and a carbohydrate binding module (*R*gNanH-CBM40), which recognizes the Neu5Ac unit (Tailford et al., 2015b). *Rg*NanH revealed a clear preference for α2,3 over α2,6-linked sialic acid. The enzyme showed a very high affinity for 3′SL, with a very low K_M_ value and high activity with an estimated k_cat_ of 25.7 min^−1^. In contrast, no enzymatic activity was observed in the presence of 6′SL (Tailford et al., 2015b). Using a high-performance anion-exchange chromatography with pulsed amperometric detection (HPAEC–PAD), it was possible to monitor the substrate consumption and product production after *Rg*NanH enzymatic reaction, and surprisingly, after the hydrolysis of 3′SL by *Rg*NanH, no free Neu5Ac was observed on the reaction products. Through ^1^H-NMR, it was elucidated that *Rg*NanH produces 2,7-anhydroα-*N*-acetylneuraminic acid (2,7-anhydro-Neu5Ac) instead of free Neu5Ac, as a hydrolysis product. The presence of two ^1^H-NMR signals at 4.56 and 4.45 p.p.m., characteristic of the protons H6 and H7 of 2,7-anhydro-Neu5Ac, unequivocally determined the hydrolysis product of *Rg*NanH (Figure 5A) (Tailford et al., 2015b). In addition, Juge and coworkers were able to obtain the X-ray structure of the *Rg*NanH catalytic domain (*Rg*NanH-GH33) complexed with 2,7-anhydro-Neu5Ac (PDB ID: 4 × 4A), since the soaking of *Rg*NanH-GH33 crystals with 3′SL resulted in the substrate turnover to the 2,7-anhydro-Neu5Ac product. These results revealed that *Rg*NanH encodes an intramolecular *trans*-sialidase responsible to selectively produce the 2,7-anhydro-Neu5Ac product from α2,3-linked Neu5Ac substrates (Tailford et al., 2015b).

The mechanism of recognition of 2,7-anhydro-Neu5Ac by *Rg*NanH-GH33 was used by Angulo and coworkers to design the differential epitope mapping by the STD methodology, the DEEP–STD NMR (Monaco et al., 2017). This method produces differential epitope maps depending on the setup of STD parameters and the experimental conditions of the sample. Basically, through the analysis of the STD–NMR experiments carried out at two irradiation frequencies, such as the aromatic region (∼6.55 p.p.m.) versus the aliphatic region (∼0.6 p.p.m.), and by changing the solvent of the sample under study (D_2_O versus H_2_O), it was possible to obtain a differential epitope map between the two conditions determined by the DEEP–STD factor (DSTD). Using this method, negative DSTDs (0.6/6.55 p.p.m.) for H8 and H9s protons of 2,7-anhydro-Neu5Ac were obtained (Figure 5B), which suggests the vicinity of these protons to *Rg*NanH-GH33 aromatic protons. In addition, the positive DSTD (0.6/6.55 p.p.m.) for CH_3_ and H3_ax_ protons indicates, in this case, the proximity to the aliphatic side chains. These results coincide with the crystal structure deduced for the complex *Rg*NanH-GH33/2,7-anhydro-Neu5Ac, where H8 and H9s of 2,7-anhydro-Neu5Ac are pointing toward the aromatic side chain of W698 of *Rg*NanH-GH33, while CH_3_ and H3_ax_ protons are in close contact with the I258, I338, and V502 amino acid side chains (Figure 5B) (Tailford et al., 2015b). The same method can also be used to discriminate the polarity of protein residues near to the ligand by recording two equal STD–NMR experiments but in different solvents, D_2_O versus H_2_O. In this case, negative DSTDs (D_2_O/H_2_O) are displayed for the H3_ax_/H3_eq_ and H9s of 2,7-anhydro-Neu5Ac, which agree with the fact that these protons are near to a highly polar region in the *Rg*NanH-GH33 binding site, in particular to a region rich in arginines (R257, R276, R575, and R637) (Figure 5C) (Tailford et al., 2015b; Monaco et al., 2017). Thus, DEEP–STD NMR allows obtaining clues concerning the putative orientation of the ligand at the binding site and the nature of amino acids involved in the recognition interface. This ligand-detected approach requires a prior knowledge on the amino acid composition of the protein’s binding site, and this information is not always readily available since it is provided from the previous experimental data obtained by NMR, X-ray, or homology modeling.

The dissection of *trans*-sialidase *Rg*NanH structure and the molecular recognition of the carbohydrate binding module *Rg*NanH-CBM40 were extensively studied by the integration of glycan arrays, STD–NMR, and X-ray crystallography (Owen et al., 2017). The isolated *Rg*NanH-CBM40 binds to most of the Neu5Ac, Neu5Gc, Neu5,9Ac2, and 2-keto-3deoxynonulosonic acid (Kdn) terminals attached with α2,3, α2,6, and α2,8 linkages. It is to be noted that the glycan microarrays for *Rg*NanH-CBM40 showed a preference for the terminal Neu5Ac over Neu5Gc and for α2,3 linkages in comparison to α2-6 and α2-8 (preference order: α2,3 ≫ α2,6 > α2,8). The STD–NMR experiments show that the sialic acid unit is required for binding *Rg*NanH-CBM40, and the *N-*acetyl methyl group is intimately in contact with the protein since it receives high STD enhancement. This result perfectly matches with the X-ray crystal structure of the complex (PDB ID: 4 X 4A), where it is possible to observe that the NHAc group is pointing toward a hydrophobic pocket (side chains of I95, Y116, and Y210) (Owen et al., 2017). The individual involvement of the arginine dyad (R204/R128) implicated in electrostatic interactions with the carboxylate group of Neu5Ac and the hydrophobic pocket (I95/Y116/Y210) involved in the recognition of NHAc at C5 of Neu5Ac was demonstrated by mutagenesis and isothermal titration calorimetry (ITC). Single or double mutations of R204 and R128 abolished the 3′SL and 6′SL binding to the *Rg*NanH-CBM40. In the hydrophobic pocket, only the I95 mutation was tolerated and did not preclude the binding (Owen et al., 2017). In summary, the full-length intramolecular *trans*-sialidase *Rg*NanH composed by a catalytic domain (*Rg*NanH-GH33) and a carbohydrate binding module (*R*gNanH-CBM40) is specific for α2,3-linked substrates to form 2,7-anhydro-Neu5Ac (Tailford et al., 2015b). However, the carbohydrate binding module (*R*gNanH-CBM40) is able to bind both α2,3 and α2,6 linked sugars (Owen et al., 2017), suggesting an additional function.

*R. gnavus* uses sialic acid as a carbon source, but before sialic acid can be metabolized, the sialic acid derivatives need to be taken into the bacterial cell. The *R. gnavus* ATCC 29149 *nan* cluster contains a single ABC transporter, two permeases, and *Rg*SBP. The *Rg*SBP subunit specifically recognizes 2,7-anhydro-Neu5Ac with a K_D_ of 2.42 ± 0.27 μM and does not bind Neu5Ac. Once again, DEEP–STD NMR was used to gain structural information and to elucidate the orientation of 2,7-anhydro-Neu5Ac in the *Rg*SBP binding pocket. DEEP–STD NMR showed that H4, H6, H7, H8, and H9’ of 2,7-anhydro-Neu5Ac are in closer contact with aromatic residues, while H3 and CH3 are oriented toward aliphatic residues (Bell et al., 2019). Then, inside the bacteria, 2,7-anhydro-Neu5Ac is converted back into Neu5Ac by *Rg*NanOx, an oxidoreductase that catalyzes the conversion of 2,7-anhydro-Neu5Ac into Neu5Ac (Figure 5A) (Bell et al., 2019). In addition, Juge and coworkers recently explained the enzymatic mechanism of *Rg*NanOx by simple analysis of 1-D and 2-D NMR spectra (Bell et al., 2020). Through the analysis of the 2-D ^1^H,^13^C HSQC spectra of 2,7-anhydro-Neu5Ac (substrate of *Rg*NanOx), Neu5Ac (*Rg*NanOx reaction product), and the reaction mixture, it was possible to identify an additional set of cross-peaks in the reaction mixture. These new sets of cross-peaks correspond to a 4-keto-2-deoxy-2,3-dehydro-*N-*acetylneuraminic acid structure. Thus, by NMR, it was possible to elucidate that the reversible conversion of 2,7-anhydro-Neu5Ac to Neu5Ac occurs through the formation of an intermediate and NAD1 regeneration (Bell et al., 2020).

In summary, *R. gnavus* expresses an intramolecular *trans*-sialidase (*Rg*NanH) that is produced from the α2,3-linked sialic acid substrates of the 2,7-anhydro-Neu5Ac analogue instead of Neu5Ac (Tailford et al., 2015b). Curiously, *R. gnavus* is the only strain, reported so far, that produces the 2,7-anhydro-Neu5Ac derivative in the gut. This mechanism to transport and convert 2,7-anhydro-Neu5Ac to Neu5Ac certainly gives *R. gnavus* a nutritional advantage. In fact, the recognition, degradation, and alterations of the host mucosal glycans by the gut microbiota have been attributed to diverse intestinal chronic diseases, such as inflammatory bowel and Crohn’s diseases (Tailford et al., 2015a; Martens et al., 2018; Henke et al., 2019). These events are a major paradigm to understand the crosstalk established between the commensal bacteria and the human host mucosal. In summary, the integration of NMR and X-ray derived data, in combination with other biophysical techniques, has contributed to provide the preliminary structural clues of the intestinal bacteria/host crosstalk that can be the first cornerstones for the development of new strategies to fight intestinal chronic diseases.

## Conclusion and Prospects

Due to their structure, location, and extensive distribution, sialic acids interact with distinct glycan-binding receptors, expressed in human cells and pathogens (viruses and bacteria), regulating an enormous diversity of physiological and pathological events. The discovery of strategies to potentially modulate sialic acid-receptor interactions in diseases is an intense research topic for many glycoscientists. In this context, the conformation and dynamics of sialoglycans in solution, together with the molecular recognition of these molecules by distinct receptors, were extensively reviewed in this article. The contribution of the concerted application of NMR and molecular modeling was especially highlighted. Nevertheless, depending on the biological system, X-ray crystallography derived data were also described, pointing out how these two techniques can complement each other. Particularly, the combination of STD–NMR and X-ray crystallography to investigate sialoglycan recognition by virus and bacteria’ receptors is a pivotal example of the complementarity of the two techniques. Even though X-ray crystallography is still considered the reference technique for obtaining high resolution glycan-receptor complexes, there are still drawbacks in the protocols for refining the glycan electron density to obtain the correct conformation (Agirre, 2017; Frenz et al., 2019). The intrinsic flexibility of sialoglycans and the tendency to form amorphous solids make them unsuitable for the X-ray crystallography technique. Therefore, NMR is an alternative technique providing unique data relative to the sialoglycan’s conformation in a free and bound state. Typical NOESY and TOCSY experiments in the absence or presence of the receptor are sufficient to ascertain the bioactive conformation of medium size sialoglycans (three and four sugar units). Additionally, progress in NMR-based approaches has been contributing to break the signal degeneracy of complex sialylated *N-*glycans (Canales et al., 2017) and to detect rapid exchanging hydroxyl groups in water, thereby helping characterize the H-bond network in PolySia homopolymers (Novakovic et al., 2021). These methodological advances pave the way to decipher the conformation and dynamics of sialoglycans with increased complexity and in different presentation contexts (intact glycoproteins and lipids) or environments (macromolecular crowding milieu). In fact, this structural information is still lacking, and it is crucial to get a more holistic view of sialoglycan’s structure and sialoglycan-protein interactions in the cellular environment. Furthermore, structural information with atomic details is still required to unravel the fine specificity of sialic-acid binding receptors and to elucidate which chemical modifications are needed in a sialoglycan molecule to increase selectivity and prevent cross reactivity. In this field, the integration of NMR and X-ray crystallographic data is a great advantage and is the logical approach to fully decode the recognition mechanism of sialoglycans by human and pathogen receptors at the molecular level. This progress is vital to understanding how sialoglycans mediate distinct binding processes in health and disease and is essential for a better-targeted design of sialic acid-based therapeutics against cancer and infection processes.
